# *Saxifraga zhiminiae* (Saxifragaceae), a new species from Xizang, China

**DOI:** 10.3897/phytokeys.274.183653

**Published:** 2026-04-30

**Authors:** Xin-Jian Zhang, Ming-Xu Zhao, Xin-Xin Zhu, Bo Xu

**Affiliations:** 1 State Key Laboratory of Plant Diversity and Specialty Crops, Kunming Institute of Botany, Chinese Academy of Sciences, Kunming, Yunnan, China College of Life Sciences, Xinyang Normal University Xinyang China; 2 Asian Elephant Research Center, National Forestry and Grassland Administration, Kunming, 650031, Yunnan, China State Key Laboratory of Plant Diversity and Specialty Crops, Kunming Institute of Botany, Chinese Academy of Sciences Kunming China; 3 Southwest Institute of Survey and Planning, National Forestry and Grassland Administration, Kunming, 650031, Yunnan, China College of Biological and Food Engineering, Southwest Forestry University Kunming China; 4 College of Life Sciences, Xinyang Normal University, Xinyang, Henan, 464000, China Asian Elephant Research Center, National Forestry and Grassland Administration Kunming China; 5 College of Biological and Food Engineering, Southwest Forestry University, Kunming, China Southwest Institute of Survey and Planning, National Forestry and Grassland Administration Kunming China

**Keywords:** New species, *Saxifraga* sect. *Ciliatae*, Saxifragaceae, taxonomy, Xizang

## Abstract

*Saxifraga zhiminiae*, a remarkable new species of the genus *Saxifraga* sect. *Ciliatae* (Saxifragaceae), is described from Xizang, China. This new species morphologically differs from all known species of sect. *Ciliatae* by its plant glabrous (except for glandular cilia on the sepal margins), leaf blade abaxially red and petals pink with a dark red base adaxially. Morphological comparison and molecular evidence from chloroplast genomes indicate that *S. zhiminiae* belongs to sect. *Ciliatae* subsect. *Hirculoideae* and is closely related to *Saxifraga diversifolia*, but it can be easily distinguished by its glabrous habit and striking petal colouration.

## Introduction

*Saxifraga* L. is the largest genus of Saxifragaceae, comprising approximately 420 species widely distributed across the Northern Hemisphere (Carruthers et al. 2024; Zhang et al. 2024a). *Saxifraga* is taxonomically complex, with high species diversity and remarkable morphological variations (Tkach et al. 2015). Gornall (1987) proposed a revised classification of *Saxifraga* with 15 sections, 19 subsections and 34 series. Recent molecular phylogenetic studies have revealed at least 13 sections and nine subsections within the monophyletic genus (Tkach et al. 2015). Sect. *Ciliatae* Haw. is the most species-rich section of *Saxifraga*, comprising ca. 200 species and is widely distributed across the Tibeto–Himalayan Region, with its highest species diversity occurring in south-western China (Yuan et al. 2023).

In China, *Saxifraga* comprises about 230 species, including several recently reported new species and resurrected species (Gao et al. 2018; Zhang et al. 2021; Zhang et al. 2023a; Zhang et al. 2023b; Zhang et al. 2024b). Sect. *Ciliatae* represents the major component of the biodiversity of *Saxifraga* in China (Pan et al. 2001). A recent molecular study has indicated that sect. *Ciliatae* is monophyletic, within which five subsections are currently recognised (Yuan et al. 2023).

The new species described here first came to our attention in August 2025 during a field survey in Xizang, China. We initially recognised it as a member of sect. *Ciliatae* because of its leaves with a glaucous abaxial surface and a prominent submarginal vein, corresponding to morphological features of key 5 of sect. *Ciliatae* (Pan et al. 2001). However, the new species is distinguished by its glabrous plant and pink petals with a dark red base adaxially. This combination of features is not found in any known species of sect. *Ciliatae*. After careful morphological examination and relevant literature reviews, we confirmed the distinctiveness of this taxon. Here, we describe the new species and provide both morphological and molecular evidence to support its taxonomic status and phylogenetic relationships.

## Materials and methods

### Morphological examinations

Ten living individuals of the new species were collected for field observations and morphological description. Voucher specimens of our collections were deposited in the Herbarium of the Kunming Institute of Botany, Chinese Academy of Sciences (KUN), Kunming, China. Morphological comparisons were conducted with herbarium specimens of sect. *Ciliatae* from CDBI, IBSC, KUN and PE (Thiers 2026), either by examining specimens directly or through digital images available via the National Plant Specimen Resource Center (www.cvh.ac.cn/index.php) and JSTOR Global Plants (https://plants.jstor.org/).

### Phylogenetic reconstruction

Leaf materials from three individuals of the new species were collected from the type locality in October 2025. Since the morphological features of the new species support its placement in sect. *Ciliatae*, which has been confirmed as monophyletic (Yuan et al. 2023), our taxon sampling focused on sect. *Ciliatae*, with species from other genera of Saxifragaceae included as outgroups. Samples of sect. *Ciliatae* species were newly sequenced here or obtained from GenBank. The final sequencing data comprised 49 accessions representing 47 species of sect. *Ciliatae*, with *Micranthes pallida* (Wall. ex Ser.) Losinsk. from *Micranthes* Haw. as outgroup. Voucher information and GenBank accession numbers were presented in Table 1.

**Table 1.** Voucher information and GenBank accessions for phylogenetic analysis.

Taxon	Voucher	GenBank accession
*Saxifraga aristulata* Hook.f. & Thomson	Chen2012223 (HNWP)	NC_070440
*Saxifraga atuntsiensis* W.W.Sm.	Chen2014542-1 (HNWP)	NC_070441
*Saxifraga gyalana* C.Marquand & Airy Shaw	Gao2018154-4 (HNWP)	NC_070469
*Saxifraga drabiformis* Franch.	Chen06194 (HNWP)	NC_070457
*Saxifraga glacialis* Harry Sm.	Chen2014165-3 (HNWP)	NC_070466
*Saxifraga balfourii* Engl. & Irmsch.	Chen2012076-5 (HNWP)	NC_070444
*Saxifraga gemmipara* Franch.	Chen2013529 (HNWP)	NC_070465
*Saxifraga brunonis* Ser.	Chen2014448 (HNWP)	NC_070449
*Saxifraga wardii* W.W.Sm.	Chen2014313-1 (HNWP)	NC_070525
*Saxifraga hispidula* D.Don	Chen2014450-5 (HNWP)	NC_070473
*Saxifraga consanguinea* W.W.Sm.	Gao2018118-1 (HNWP)	NC_070454
*Saxifraga nangxianensis* J.T.Pan	Chen2014410-4 (HNWP)	NC_070492
*Saxifraga hemisphaerica* Hook.f. & Thomson	Gao2018125-4 (HNWP)	NC_070471
*Saxifraga sediformis* Engl. & Irmsch.	Chen2012104-2 (HNWP)	NC_070506
*Saxifraga sanguinea* Franch.	Chen2014234-5 (HNWP)	NC_070505
*Saxifraga umbellulata* Hook.f. & Thomson	Chen2013298 (HNWP)	NC_070518
*Saxifraga auriculata* Engl. & Irmsch.	Chen06112 (HNWP)	NC_070443
*Saxifraga egregia* Engl.	Gao2018170-1 (HNWP)	NC_070459
*Saxifraga tigrina* Harry Sm.	LiuJQ-09XZ-354 (KUN)	PZ168315
*Saxifraga parnassifolia* D.Don	HL040 (KUN)	PZ168311
*Saxifraga hypericoides* Franch.	Chen2012142-2 (HNWP)	NC_070475
*Saxifraga litangensis* Engl.	Gao2018151-9 (HNWP)	NC_070481
*Saxifraga isophylla* Harry Sm.	Chen2014314-1 (HNWP)	NC_070478
*Saxifraga subaequifoliata* Irmsch.	Chen2013265 (HNWP)	NC_070512
*Saxifraga omphalodifolia* Hand.-Mazz.	deng10109 (KUN)	PZ168313
*Saxifraga haplophylloides* Franch.	deng13036 (KUN)	PZ168312
*Saxifraga implicans* Harry Sm.	Chen2012137-1 (HNWP)	NC_070476
*Saxifraga stellariifolia* Franch.	Chen06089 (HNWP)	NC_070511
*Saxifraga diversifolia* var. *angustibracteata* (Engl.& Irmsch.) J.T.Pan	Chen03107 (HNWP)	NC_070456
*Saxifraga subamplexicaulis* Engl. & Irmsch.	deng2497 (KUN)	PZ168310
*Saxifraga pardanthina* Hand.-Mazz.	Chen06243 (HNWP)	NC_070496
*Saxifraga pratensis* Engl. & Irmsch.	Chen06199 (HNWP)	NC_070500
*Saxifraga erectisepala* J.T.Pan	Chen06154 (HNWP)	NC_070460
*Saxifraga cardiophylla* Franch.	DT078-9 (KUN)	PZ168314
*Saxifraga diversifolia* Wall. & Ser.	Chen2013522 (HNWP)	NC_070455
*Saxifraga tibetica* Losinsk.	Gao2018123-4 (HNWP)	NC_070516
*Saxifraga tangutica* Engl.	Gao2018115-2 (HNWP)	NC_070514
*Saxifraga kingdonii* C.Marquand	Chen2013469 (HNWP)	NC_070479
*Saxifraga lychnitis* Hook.f. & Thomson	Gao2018152-4 (HNWP)	NC_070482
*Saxifraga insolens* Irmsch.	Chen2012115-3 (HNWP)	NC_070477
*Saxifraga maxionggouensis* J.T.Pan	Chen2012218-4 (HNWP)	NC_070484
*Saxifraga chumbiensis* Engl. & Irmsch.	Chen2014505-4 (HNWP)	NC_070451
*Saxifraga tsangchanensis* Franch.	Chen2013330 (HNWP)	NC_070517
*Saxifraga nigroglandulifera* N.P.Balakr.	Chen2012196-1 (HNWP)	NC_070493
*Saxifraga eglandulosa* Engl.	Chen2014461-4 (HNWP)	NC_070458
*Saxifraga moorcroftiana* (Ser.) Wall. & Sternb.	Chen2014510-2 (HNWP)	NC_070488
*Saxifraga zhiminiae*	Tsui-4759A (KUN)	PZ168316
*Saxifraga zhiminiae*	Tsui-4759C (KUN)	PZ168317
*Saxifraga zhiminiae*	Tsui-4759B (KUN)	PZ168318
*Micranthes pallida* (Wall. ex Ser.) Losinsk.	Chen2014429-3 (HNWP)	NC_070489

Phylogenetic analyses were conducted using genome skimming data. Total genomic DNA was extracted from leaf material and library construction and sequencing were carried out at Novogene (Beijing, China) using the Illumina HiSeq 4000 platform with 150 bp paired-end reads. Plastid genomes were assembled using the GetOrganelle pipeline (Jin et al. 2020) and annotated in batches using GeSeq (https://chlorobox.mpimp-golm.mpg.de/geseq.html) (Tillich et al. 2017). Shared protein-coding genes (PCGs) of the plastid genome were concatenated and aligned in MACSE v.2 and trimmed by Gblocks 0.91b implemented in Phylosuite v.1.2.3 (Talavera and Castresana 2007; Ranwez et al. 2018; Zhang et al. 2020). Phylogenetic reconstructions were performed using both Maximum Likelihood (ML) and Bayesian Inference (BI) methods. The optimal substitution model (GTR+I+G) was selected using jModelTest v.2.1.7 (Posada 2008). ML analysis was conducted in IQ-TREE v.3.0.1 with 1000 bootstrap replicates to estimate clade support (Wong et al. 2025). BI analysis was carried out in MrBayes v.3.2 (Ronquist et al. 2012), with four parallel MCMC chains run for 20 million generations, sampled every 1000 generations and the first 25% of samples discarded as burn-in. Convergence was considered achieved when the average standard deviation of split frequencies fell below 0.01 (Ronquist et al. 2012).

## Results

### Morphological comparison

**Figure 1.**
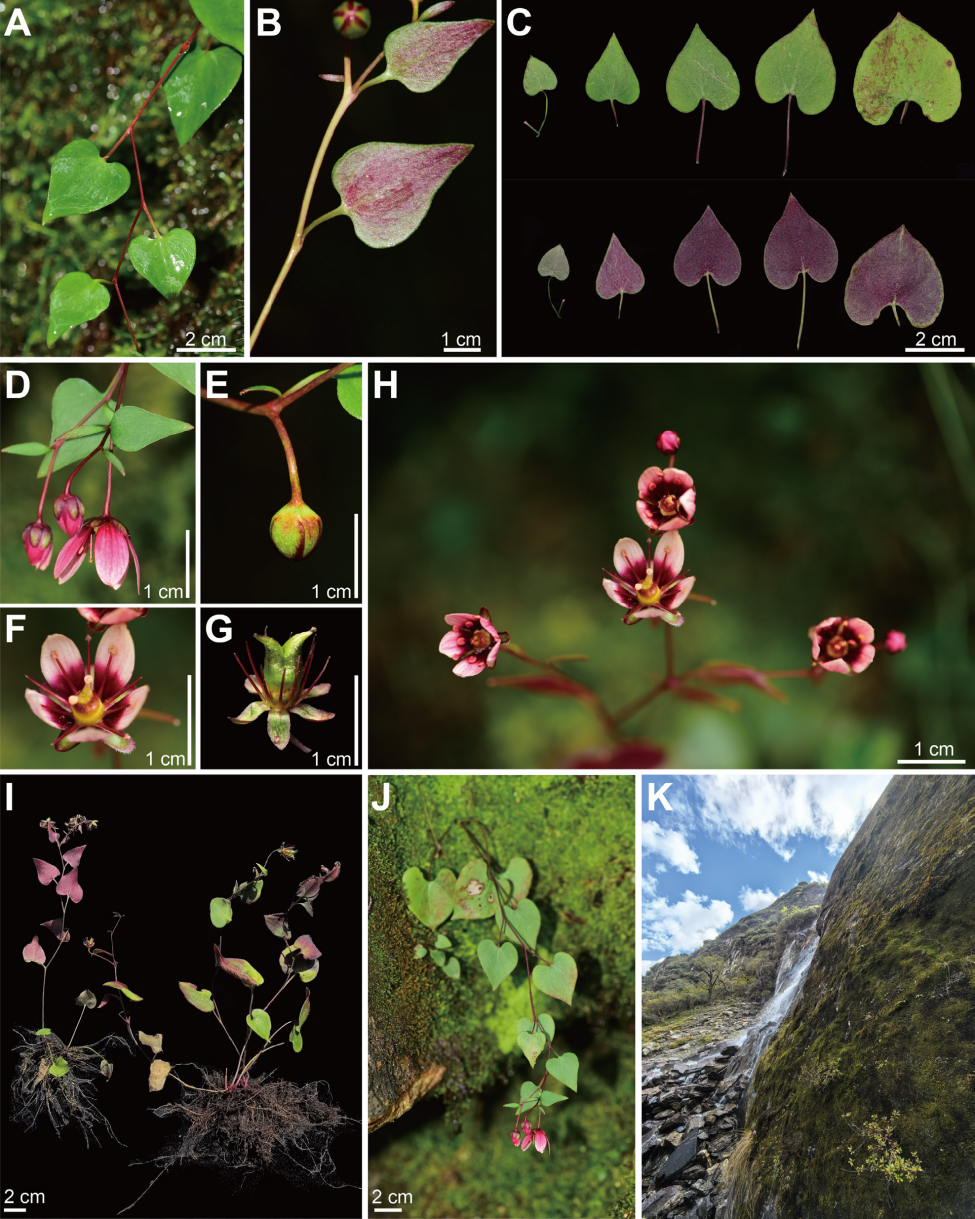
*Saxifraga zhiminiae*. **A–C**. Leaf blade with petiole and stem glabrous; **D**. Inflorescence; **E**. Flower bud with glabrous pedicel; **F, H**. Flower with a dark red base adaxially; **G**. Fruit; **I**. Plant; **J**. Habit; **K**. Habitat (**G, I, K**, photo by Bo Xu; all other images by Xinxin Zhu).

**Figure 2.**
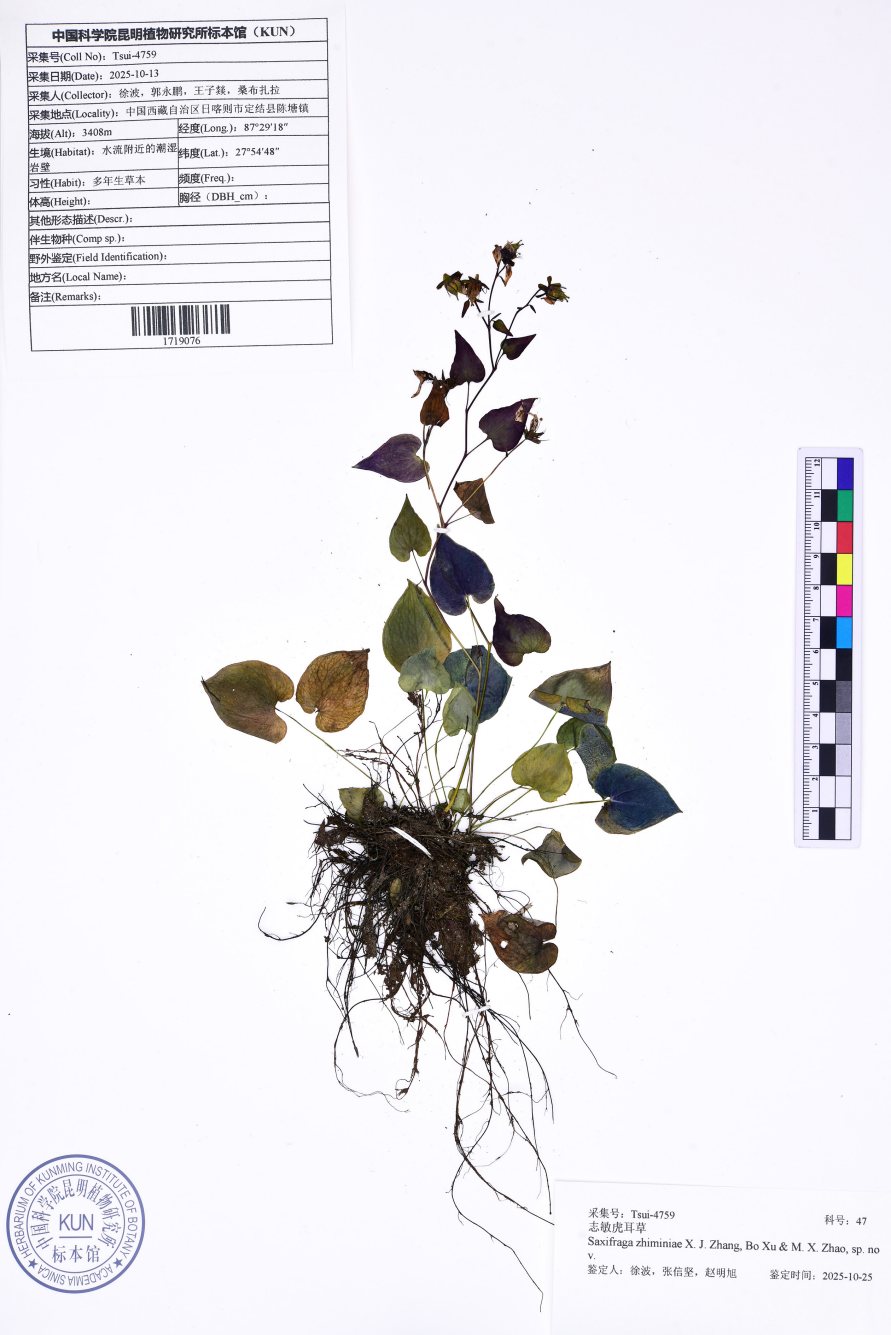
Photograph of the holotype of *Saxifraga zhiminiae*. (Tsui-4759, KUN1719076).

The leaves of the new species have a glaucous abaxial surface and a prominent submarginal vein, which indicates a morphological affinity with species included in key 5 of sect. *Ciliatae* (Pan et al. 2001). Morphological comparisons with known species in key 5 of sect. *Ciliatae* indicate that the new species resembles *Saxifraga diversifolia* Wall. & Ser. by having leaf blades with a prominent submarginal vein, basal leaves with a long petiole, ovate-cordate leaf blades with a cordate base and an entire margin (Figs 1, 2). However, the new species differs from *S. diversifolia* in being glabrous, except for glandular cilia along the sepal margins (vs. stems and pedicels glandular-hairy), having pink petals with a dark red base on the adaxial surface (vs. yellow petals), possessing leaf blades that are red abaxially (vs. green abaxially) and having sepals that are erect to spreading (vs. reflexed). Phylogenetic analyses indicate that the new species is sister to *Saxifraga moorcroftiana* (Ser.) Wall. & Sternb. However, the new species is distinguishable from the latter by its glabrous habit (versus stem brown crisped glandular villous), cauline leaves with a petiole (versus cauline leaves usually sessile) and petals pink with a dark red base adaxially (versus petals yellow). The morphological comparisons amongst the new species, *S. diversifolia* and *S. moorcroftiana* are presented in Table 2.

**Table 2.** Diagnostic characters of *Saxifraga zhiminiae* and comparison with *S. diversifolia* and *S. moorcroftiana*.

Characters	*Saxifraga zhiminiae*	*S. diversifolia*	*S. moorcroftiana*
**Trichomes on Stems**	absent	proximally crisped villous or glabrous, distally shortly glandular hairy	proximally brown crisped glandular villous, glabrous in median part, distally brown glandular pilose.
**Leaf shape**	subcordate or ovate-cordate	subcordate or ovate-cordate to narrowly ovate	pandurate-elliptic to oblong, semi-amplexicaul
**Trichomes on leaf**	absent	abaxially and marginally brown pubescent	abaxially and marginally brown glandular pilose
**Trichomes on petiole**	absent	brown crisped villous	sparsely brown glandular villous
**Inflorescence**	glabrous	brown glandular hairy	dark purple glandular hairy
**Sepals**	erect to spreading, glabrous, except for ciliate in margin	reflexed, abaxially and margin glandular hairy	erect to spreading, abaxially and margin glandular hairy
**Petals**	adaxially pink with a dark red base, abaxially red	yellow on both surfaces	yellow on both surfaces

### Phylogenetic reconstruction

**Figure 3.**
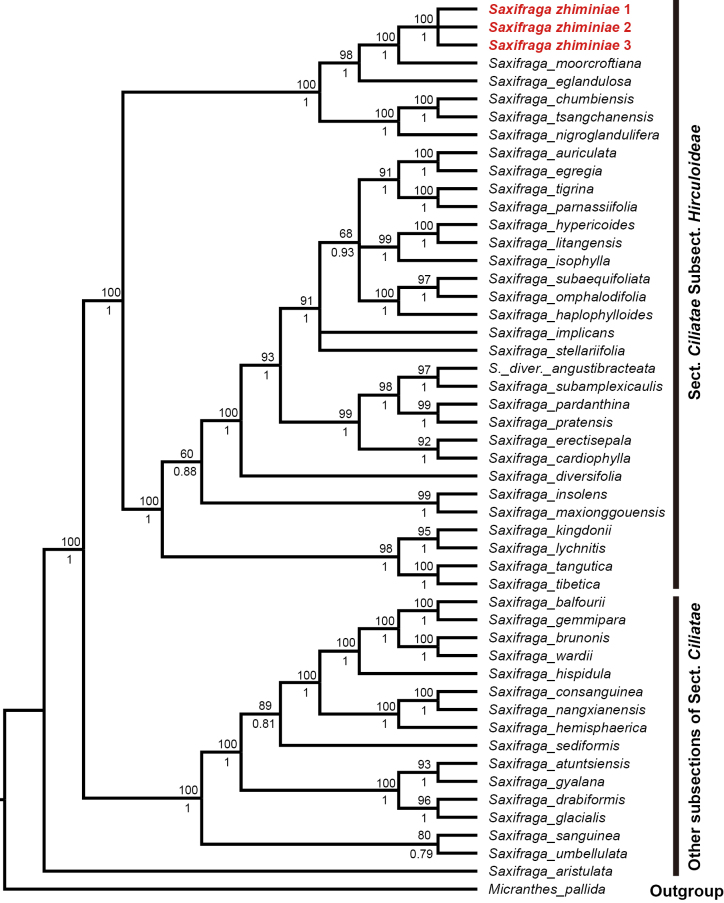
The phylogenetic position of *Saxifraga zhiminiae* in *Saxifraga* sect. *Ciliatae* derived from the combined 69 PCGs of the plastid genome, with *Micranthes pallida* as outgroup. Numbers above branches indicate ML bootstraps, numbers below branches are Bayesian posterior probability.

A total of 47 taxa were included in the phylogenetic analysis using 69 shared protein-coding genes (PCGs) from the plastid genome of 50 samples. The resulting concatenated matrix was 67,734 bp in length after alignment. The 50% majority-rule consensus tree (Fig. 3) generated from the ML and BI analyses showed that three samples of the new species grouped together (ML = 100, BI = 1) and were nested within subsection *Hirculoideae* Engler & Irmscher. The phylogenetic analyses further revealed a sister relationship between the new species and *S. moorcroftiana* with strong support (ML = 100, BI = 1).

### Taxonomic treatment

#### Saxifraga zhiminiae

Taxon classification

Plantae

SaxifragalesSaxifragaceae

X.J.Zhang, Bo Xu & M.X.Zhao sp. nov.

7188FA95-9566-5664-8693-0D15C0E9AF8F

urn:lsid:ipni.org:names:77379400-1

Figs 1, 2

##### Type.

China • Xizang: Xigaze City, Dinggyê County, Chentang Town, 27°54'48"N, 87°29'18"E, alt. 3408 m, 13 October 2025, *Bo Xu, Yongpeng Guo, Ziyi Wang, Sangbu Zhala Tsui-4759* (holotype: KUN1719076!; isotypes: KUN1719075!, SWFC!).

##### Diagnosis.

*Saxifraga zhiminiae* resembling *S. diversifolia* and *S. moorcroftiana*, but the species is easily distinguished from the latter two and any other species of *Saxifraga* sect. *Ciliatae* by its plant glabrous, except for glandular-ciliate sepals, leaf blade abaxially red, petals adaxially pink or whitish, proximally dark red and abaxially red.

##### Description.

Herbs perennial, 15–30 cm tall. Stem glabrous. Basal leaves with petiole 3–6 cm, glabrous; leaf blade cordate to ovate-cordate, 1.8–4.0 × 1.2–3.0 cm, both surfaces and margin glabrous, adaxially green, abaxially green to purplish-red, base cordate, apex acuminate. Cauline leaves 8–12; petiole 0.6–3.5 cm, glabrous; leaf blade subcordate or ovate-cordate, 1.2–4.2 × 0.6–3.1 cm, base cordate or subcordate, apex acuminate; proximal leaf blades larger, both surfaces and marginally glabrous or subglabrous; distal leaf blades smaller, usually glabrous on both surfaces and margin. Cyme corymbose, 4–8 cm, 3–7-flowered; pedicels 8–20 mm, glabrous. Sepals erect to spreading, narrowly ovate, 4.0–5.5 × 1.2–2.3 mm, purplish-red or greenish, glabrous on both surfaces, veins (3) –5, margin glandular ciliate, apex obtuse or acute. Petals adaxially pink or whitish, proximally dark red, abaxially red, apex whitish, elliptic to obovate, 6–10 × 3–5 mm, usually not callose, (3–) 5 (–7)-veined, base narrowed into a short claw, apex obtuse. Stamens 5–8 mm long, filaments linear, dark red with a green base, anther yellowish-orange. Ovary superior, ovoid, 5–8.2 mm; styles 1–2 mm. Seeds elliptic, slightly curved at both ends, ca. 0.5 mm long.

##### Phenology.

Flowering and fruiting from August to October, based on field observations and herbarium specimens.

##### Distribution and ecology.

*Saxifraga zhiminiae* is currently known only from Chentang Town, Dinggyê County, Xizang, China, where it grows on moist cliff faces near waterfalls at elevations of 3000–3500 m.

##### Etymology.

*Saxifraga zhiminiae* is characterised by its attractive, red cordate leaves and striking petal colouration. The specific epithet honours Professor Zhimin Li of Yunnan Normal University, who has dedicated her career to botanical education. With unwavering commitment, she has inspired and nurtured numerous generations of botanists.

## Discussion

The new species has basal leaves with a long petiole and leaf blades characterised by a prominent submarginal vein. These features indicate its position in key 5 of *Saxifraga* sect. *Ciliatae* (Pan et al. 2001). Morphologically, the species resembles *S. diversifolia* and *S. moorcroftiana* in overall plant architecture. However, it is readily distinguished from both species by its entirely glabrous plant and petals pink with a dark red base adaxially. This distinctive combination of characters is not observed in any other currently recognised member of sect. *Ciliatae*. We consider these morphological differences sufficient to support its recognition as a distinct species.

Phylogenetic analyses, based on plastid genomes, support the phylogenetic position of *S. zhiminiae* in subsect. *Hirculoideae* and recover it as sister to *S. moorcroftiana*. Sect. *Ciliatae* represents the largest section of *Saxifraga* and subsect. *Hirculoideae* is the most species-rich and taxonomically complex subsection amongst the currently recognised five subsections of sect. *Ciliatae*. Based on morphological characteristics, subsect. *Hirculoideae* has been divided into three species complexes, with taxa exhibiting a prominent submarginal vein assigned to the *S. diversifolia* complex (Ma et al. 2022). Although the prominent submarginal vein suggests that the new species belongs to key 5 of *Saxifraga* sect. *Ciliatae*, corresponding to the *S. diversifolia* complex, its glabrous plant and distinctive petal colouration clearly differentiate it from all currently recognised members of this taxonomically difficult group (Pan et al. 2001; Ma et al. 2022). The discovery of this morphologically unusual taxon underscores the likelihood that species diversity within this species-rich and taxonomically complex group remains underestimated. Continued field exploration and phylogenetic work will be essential to elucidate patterns of morphological variation and refine species delimitation in subsect. *Hirculoideae*. At present, *S. zhiminiae* is known only from Chentang Town, Dinggyê County, Xizang, China. This region was historically regarded as China’s last ‘land-locked island’, with vehicular access was not established until late 2017. Consequently, its biodiversity remains insufficiently documented and warrants further investigation.

## Supplementary Material

XML Treatment for Saxifraga zhiminiae

## Additional information

### Artificial Intelligence (AI) use

The authors accept full responsibility for the content of the manuscript, including the disclosure of any use of AI.

No AI tools were used in the preparation of this manuscript.

### Author ORCIDs

X.-J. Zhang https://orcid.org/0000-0002-0668-8497

M.-X. Zhao https://orcid.org/0000-0002-4981-9792

X.-X. Zhu https://orcid.org/0000-0002-9252-7453

B. Xu https://orcid.org/0000-0002-1473-9258

### Data availability

All of the data that support the findings of this study are available in the main text. The newly sequenced data of this study have been deposited in The National Center for Biotechnology Information (NCBI) database. GenBank accession numbers of the sequencing data can be found in Table 1.
